# Evolution and genetic diversity of the Spain^23F^-ST81 clone causing adult invasive pneumococcal disease in Barcelona (1990–2012)

**DOI:** 10.1093/jac/dkt473

**Published:** 2013-12-08

**Authors:** A. Domenech, C. Ardanuy, I. Grau, L. Calatayud, R. Pallares, A. Fenoll, A. B. Brueggemann, J. Liñares

**Affiliations:** 1 Microbiology Department, Hospital Universitari de Bellvitge-IDIBELL-Barcelona University, Barcelona, Spain; 2 CIBERES (Ciber de Enfermedades Respiratorias), ISCIII, Madrid, Spain; 3 Infectious Diseases Department, Hospital Universitari de Bellvitge-IDIBELL, Barcelona, Spain; 4 Spanish Reference Pneumococcal Laboratory, Inst. Salud Carlos III, Majadahonda, Madrid, Spain; 5 Department of Zoology, University of Oxford, Oxford, UK

**Keywords:** *Streptococcus pneumoniae*, PMEN1, Spanish

## Abstract

**Objectives:**

We aimed to analyse the clinical epidemiology and genetic diversity of invasive pneumococcal disease (IPD) episodes attributed to the Spain^23F^-ST81 (PMEN1) clone.

**Methods:**

Fifty-eight (2.7%) of 2117 invasive pneumococci isolated from adult patients during the 1990–2012 period shared a PFGE pattern related to the PMEN1 clone. The genotype was confirmed by multilocus sequence typing. The *pbp2x*, *pbp1a*, *pbp2b* and *pspA* genes were PCR-amplified and sequenced. Polymorphisms in the *pspC* gene were identified by PCR restriction fragment length polymorphism. The presence of transposons with erythromycin and tetracycline resistance determinants was detected by PCR.

**Results:**

The prevalence of the PMEN1 clone increased from 0.8% in 1991 to 6.2% in 2001, and decreased to 0% in 2010–12, concomitant with the introduction of the seven-valent pneumococcal conjugate vaccine for children. A total of 93.1% of patients had pneumonia, meningitis or peritonitis; 87.9% of patients had associated underlying diseases, mainly cancer, chronic obstructive pulmonary disease and diabetes. Two closely related sequence types (STs) (ST81, *n* = 52; ST85, *n* = 6) were detected, with different serotypes: 23F (*n* = 42), 19A (*n* = 9) and 19F (*n* = 6). All the isolates were resistant to penicillin, co-trimoxazole and chloramphenicol. All the isolates also shared the same *pbp1a* allele, whereas multiple alleles of *pbp2b*, *pbp2x*, *pspA* and *pspC* were detected. Of the isolates, 89.7% were tetracycline resistant and 60.3% (*n* = 35) were macrolide resistant, and resistance was associated with different Tn*916*-like transposons.

**Conclusions:**

Adult IPD caused by this clone was mainly detected in patients with underlying conditions, and genetic variability was observed among PMEN1 isolates collected in our area over the past 20 years.

## Introduction

*Streptococcus pneumoniae* (pneumococcus) is a human pathogen responsible for a wide variety of invasive diseases, including bacteraemic pneumonia and meningitis.^1^ Since the 1970s, β-lactam susceptibility among pneumococci has generally decreased, largely as a consequence of the emergence and spread of a few multidrug-resistant clones, whose nomenclature has been standardized by the Pneumococcal Molecular Epidemiology Network (PMEN).^2^ PMEN1 (reference strain ATCC 700669), a multidrug-resistant clone, has been identified globally with different capsular types, penicillin-binding protein (PBP) profiles and macrolide resistance determinants.^3,4^ The first recognized isolate of this clone was recovered at our hospital in 1984 from a 64-year-old woman with breast cancer during an episode of bacteraemic pneumonia; the pneumonia was successfully treated with high doses of intravenous penicillin G.^5^

Penicillin resistance among invasive pneumococci increased from 6% to 44% in Spain during the 1980s, in part due to the increase of invasive multidrug-resistant serotype 23F, which accounted for 5.6% of all invasive penicillin-resistant serotypes in 1979–81 compared with 18.4% in the period 1982–84.^6^ Similar increases were observed in our hospital, where penicillin resistance rates among invasive pneumococci isolated from adult patients at Hospital Universitari de Bellvitge gradually increased from 4.3% in 1979 to 40% in 1990.^7^ A total of 23.8% of all penicillin-resistant pneumococci isolated during this period expressed serogroup 23 and were also resistant to tetracycline, chloramphenicol and co-trimoxazole. This antibiotic resistance pattern was identical to that of the PMEN1 reference strain.^7^

Resistance to β-lactam antibiotics in pneumococci is due to alterations in PBPs, especially PBPs 2X, 1A and 2B.^8^ In the late 1990s, the majority of PMEN1 isolates were also resistant to macrolides as a result of two main mechanisms: target site modification by methylases encoded by *erm*(B) (referred to as the MLS_B_ phenotype) and/or an efflux pump encoded by the *mef*(A/E) gene (referred to as the M phenotype).^9^ The frequent association of co-resistance to macrolides and tetracycline [*tet*(M)] is due to the presence of Tn*916-*like transposons.^10^ In PMEN1-like isolates, these transposons are usually integrated in an ∼81 kb integrative and conjugative element (ICE), called ICESp^23F^ST81; PMEN1-like isolates are also chloramphenicol resistant due to the presence of *cat*, which codes for a chloramphenicol acetyltransferase and is also part of ICESp^23F^ST81.^11^

Recently, two studies have used whole-genome sequencing to analyse isolates from the PMEN1 genetic lineage. The first study focused solely on the PMEN1 lineage and described considerable genomic diversity believed to have originated by horizontal gene transfer in response to antimicrobial and vaccine selective pressures.^12^ These authors also found a high sequence variability of surface-expressed proteins such as pneumococcal surface protein (Psp) A and PspC,^12^ which are candidates in protein antigen-based pneumococcal vaccines under development.^13^ The second study focused on understanding the evolution of penicillin resistance among pneumococci and revealed a surprising directional transmission of penicillin-resistance genes and other genes associated with virulence and antibiotic resistance from the PMEN1 clone to several genetically unrelated clones.^14^

We used a collection of 58 invasive PMEN1-like pneumococcal isolates recovered during the last two decades from adults in our geographical area to address several aims: (i) to evaluate the clinical epidemiology of episodes of invasive pneumococcal disease (IPD) caused by these PMEN1-like isolates; (ii) to detect changes in the molecular epidemiology of these isolates throughout the study period; and (iii) to analyse the sequence diversity among several genes associated with antimicrobial resistance or which encode surface-expressed proteins.

## Methods

### Ethics

This study and publication of the results were approved by the Comité Ètic d'Investigació Clínica del Hospital Universitari de Bellvitge.

### Study setting, bacterial isolates and antimicrobial susceptibility

This study was performed at Hospital Universitari de Bellvitge, a 1000 bed tertiary teaching hospital in Barcelona, Spain, that admits only adult patients and serves a population of about 600 000 people. The clinical data of patients with IPD were prospectively collected and recorded in a database, including data related to demographics, comorbidities and outcome for each patient.

A total of 2117 isolates were recovered from patients with IPD between 1990 and 2012, of which 58 (2.7%) unique isolates shared the same PFGE pattern as the PMEN1 clone and were thus selected for further study.

The overall description of serotypes and PFGE patterns of pneumococci isolated from cases of IPD from 1997 to 2007 was published in a previous study.^15^ Susceptibility to 22 antimicrobials (MIC) was determined by broth microdilution (STRHAE1; Sensititre, West Sussex, UK), following CLSI recommendations.^16^ *S. pneumoniae* ATCC 49619 and ATCC 700669 were used as control isolates.

### Serotyping and molecular characterization of isolates

Serotyping was performed at the Spanish Pneumococcus Reference Laboratory (Centro Nacional de Microbiología, Majadahonda, Madrid), using the Quellung reaction. Multilocus sequence typing (MLST) was performed following the standard protocol,^17^ and alleles and sequence types (STs) were assigned using the pneumococcal MLST web site (www.mlst.net).

*pbp1a*, *pbp2b* and *pbp2x* were PCR-amplified and sequenced, using primer sets and conditions described previously.^18^ Macrolide resistance genes *erm*(B), *erm*(TR) and *mef*(A/E), the tetracycline resistance determinant *tet*(M), and genes associated with the Tn*916* family of transposons (*int*, *xis*, *tnpA* and *tnpR*) were studied by PCR as previously described.^19^ Linkage analysis was also performed using the previously described primer sets J11/J12, tetM2/xis_rv and int_fw/xis_rv.^19^ The presence or absence of these genes and the linkage analysis of each isolate, plus publicly available sequences from online databases (GenBank: http://www.ncbi.nlm.nih.gov/genbank/; and ICEberg: http://db-mml.sjtu.edu.cn/ICEberg/index.php), were analysed in combination in order to predict the *Tn*916-like ICEs carried by each PMEN1-like isolate.

*pspA* was PCR-amplified and sequenced using primer sets and conditions previously described.^20,21^ Isolates with a negative *pspA* PCR were retested using a new pair of primers: *pspA*_Fw (5′-CAAGCTCTCTCATCGGAAGTGTTTT-3′) and *pspA*_Rv (5′-CATCTTCAGGATCAGCCCCTCCAAG-3′). *pspC* was characterized by restriction fragment length polymorphism analysis of PCR products (PCR-RFLP). Briefly, *psp*C was amplified as previously reported,^22^ and then the PCR products were separately digested with HinfI and MboII. After electrophoresis in 2% agarose gels, the PCR-RFLP profiles were compared between isolates.

### Genetic similarities and statistical analyses

In order to analyse the similarities between the isolates, a SplitsTree was constructed.^23^ Briefly, these data were converted into strings, using ‘1’ for positive or ‘0’ for negative results for the different variables: different allele sequences of *pspA* and *pspC* genes, serotype (23F, 19A or 19F), the presence of transposon-related genes [*erm*(B), *mef*(A), *tet*(M), *int*, *xis*, *tnpA* and *tnpR*], *pbp1a* (allele A), *pbp2b* (alleles A–E), *pbp2x* (alleles A–C) and ST (ST81 and ST85). Thus, a ‘string’ contained the complete profile of the genes analysed, and a string of the same length was obtained for every isolate. A tree was constructed using the software SplitsTree 4.10 with the following conditions: character transformation, uncorrected *P*; distance transformation, NJ; and variance, ordinary least squares. Statistical analyses were carried out using SPSS for Windows (version 18.0). We used the χ^2^ or Fisher's exact test to compare proportions. Two-sided *P* values <0.05 were considered statistically significant.

## Results and discussion

The PMEN1 clone was the first multidrug-resistant pneumococcal clone described,^2–4^ and since then it has been recovered from ill and healthy people in many geographical locations around the world (www.mlst.net); however, little information is available about the clinical and demographic data of the patients infected by PMEN1-like pneumococci.

### Patient characteristics

Fifty-eight patients with IPD had an isolate recovered from their clinical sample that matched the PFGE profile of PMEN1, and the clinical characteristics of these patients are shown in Table 1. The patients were predominantly male and the mean ± SD age was 60 ± 17 years; most patients had pneumonia (67.2%), meningitis (15.5%) or peritonitis (10.3%).

**Table 1.** Characteristics of 58 adult patients with IPD caused by the PMEN1 clone

Age (years), mean ± SD (range)	60 ± 17 (19–93)
Age group, *n* (%)	
18–65 years	33 (56.9)
>65 years	25 (43.1)
Male, *n* (%)	41 (70.7)
Positive blood culture, *n* (%)	39 (67.2)
Clinical syndrome, *n* (%)	
pneumonia	39 (67.2)
meningitis	9 (15.5)
peritonitis	6 (10.3)
bacteraemia without focus	3 (5.2)
septic arthritis	1 (1.7)
Comorbidity, *n* (%)	51 (87.9)
Main underlying diseases, *n* (%)	
cancer	14 (24.1)
chronic obstructive pulmonary disease	9 (15.5)
diabetes mellitus	9 (15.5)
cirrhosis	8 (13.8)
HIV/AIDS	7 (12.1)
cerebrovascular diseases	6 (10.3)
cardiovascular disease	5 (8.6)
chronic renal failure	1 (1.7)
Patients with shock, *n* (%)	10 (17.2)
30 day mortality, *n* (%)	15 (25.9)

Fifteen of 58 patients with PMEN1 died: 8 of 42 (19.0%) patients infected by PMEN1-like isolates that were serotype 23F, 4 of 9 (44.4%) that were serotype 19A, 2 of 6 (33.3%) that were serotype 19F and one patient infected by a non-typeable PMEN1 isolate. The differences in serotype-specific mortality rates are interesting, but should be interpreted with caution due to the small number of IPD episodes in our series. Furthermore, most of the patients also had immunosuppressive or chronic underlying diseases, which may contribute to the high mortality rate and is consistent with other reports that have suggested that serotypes 23F, 19F and 19A have a low invasive disease potential, but often cause infection in patients with underlying diseases.^24,25^ Similarly, a recent study, performed in our hospital on IPD in adults aged 18–64 years old, showed that serotype 23F was more frequently isolated from patients with comorbidities than from healthy adults.^26^

### Antibiotic susceptibility

**Figure 1.**
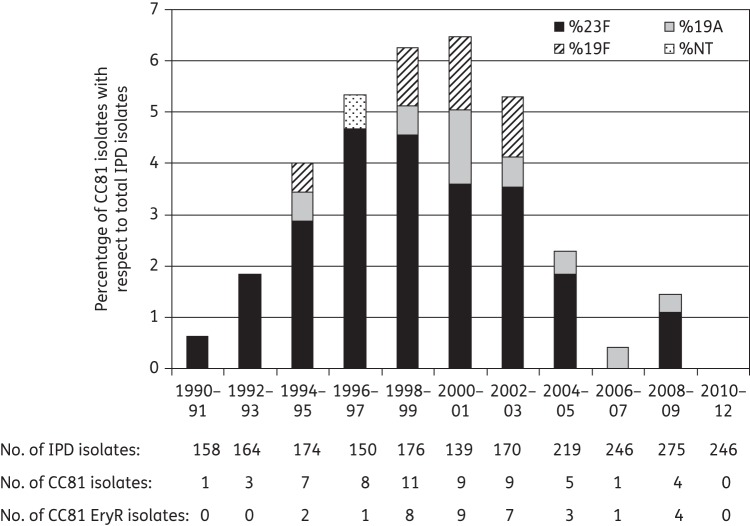
Trends in PMEN1 isolates throughout the study period. NT, non-typeable; EryR, erythromycin-resistant.

All 58 study isolates were resistant to penicillin (MIC range: 1–4 mg/L), chloramphenicol (MIC ≥8 mg/L) and co-trimoxazole (MIC ≥2/38 mg/L), and 89.7% of isolates were resistant to tetracycline (MIC ≥4 mg/L). Only one isolate, dated 2002, was resistant to levofloxacin (MIC = 16 mg/L) due to ParC (S79F) and GyrA (S81F) substitutions, as previously reported.^27^ Over half of the isolates (*n* = 35; 60.3%) were resistant to erythromycin (MIC ≥32 mg/L) and clindamycin (MIC ≥32 mg/L). Figure 1 indicates the proportion of PMEN1-like isolates identified from among all IPD isolates collected at our hospital between 1990 and 2012. The first invasive erythromycin-resistant PMEN1-like isolate was detected in 1995, but such isolates were frequently recovered after 1997. The emergence and dissemination of erythromycin-resistant PMEN1-like isolates in our hospital population coincided with an increase in the use of long-acting macrolides in Spain (clarithromycin and azithromycin were introduced in 1991 and 1992, respectively).^28^

Between 1995 and 2003, isolates belonging to the PMEN1 clone were the second most common cause of IPD in our area;^15^ however, a significant decrease (*P* < 0.05) was observed from 2004 onwards, leading to an apparent elimination of PMEN1-like isolates among IPD cases from 2010 onwards. A marked decrease in the prevalence of other multidrug-resistant clones that expressed serotypes included in the seven-valent pneumococcal conjugate vaccine (PCV7; which included serotypes 4, 6B, 9V, 14, 18C, 19F and 23F) was also detected.^15^ Overall, these decreases were possibly due to a herd immunity effect observed after the introduction of PCV7 among children in June 2001. A similar reduction was noted in a multicentre study performed in Barcelona, where PMEN1-like isolates accounted for <1% of 609 IPD episodes in 2009 in children and adults.^29^ A decrease in the prevalence of multidrug-resistant serotype 23F isolates has also been reported in other countries where PCV7 was introduced.^30,31^

### Serotypes and MLST genotypes

The most common serotype expressed by the PMEN1-like isolates was serotype 23F (*n* = 42), while the remaining isolates were of other serotypes (19A, *n* = 9; 19F, *n* = 6) or were non-typeable by Quellung and PCR (*n* = 1). Serotype variants of PMEN1 are well recognized (www.mlst.net).^12,32^ Two different, but closely related, STs were detected: 52 (89.7%) isolates were ST81, identical to the PMEN1 reference strain, and the remaining six isolates were ST85, a single-locus variant of ST81. Among the ST85 isolates, five expressed serotype 23F and one was a serotype 19F isolate.

### PBP characterization

**Figure 2.**
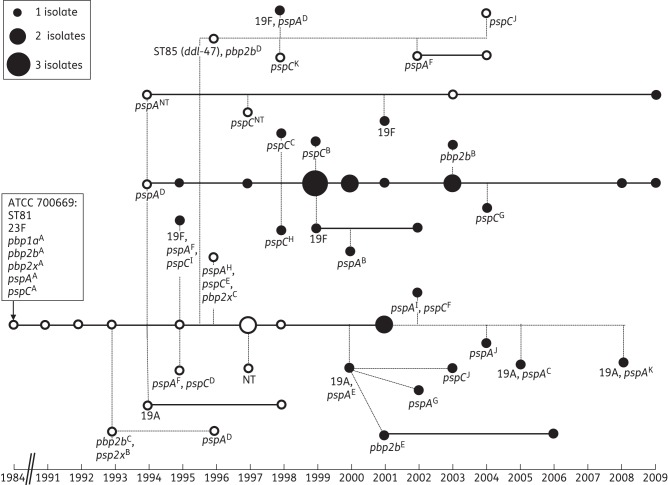
Schematic of changes occurring among PMEN1-like isolates over time, with changes marked as branches off the main line starting with the PMEN1 reference strain. White circles indicate macrolide-susceptible isolates and black circles indicate macrolide-resistant isolates. A continuous line indicates isolates with an identical serotype, *pbp* and *pspA* alleles and *pspC* polymorphisms. NT, non-typeable.

The *pbp1A* sequence was identical among all 58 isolates, whereas different alleles of *pbp2x* and *pbp2b* were detected (Figure 2). Fifty-five isolates had an identical *pbp2x* sequence (allele A) to the PMEN1 reference strain; among the remaining three isolates, all serotype 23F, two divergent sequences were identified, allele B (two isolates) and allele C, which were 1.0% and 2.8% divergent from the PMEN1 reference sequence, respectively. The sequence differences seen in alleles B and C were typical mosaic patterns of nucleotide diversity, suggestive of recombination events at this locus.

Forty-seven of 58 (81.0%) isolates had the same *pbp2b* sequence as the PMEN1 reference strain (allele A). Three isolates varied from allele A at a single nucleotide (alleles E and B), which conferred an amino acid substitution (G1189A, *n* = 2; G130A, *n* = 1; respectively). The *pbp2b* sequences of two alleles differed from that of allele A in mosaic blocks: alleles C (*n* = 2 isolates) and D (*n* = 6 isolates) were 3.3% and 9.1% divergent from allele A, respectively. The six isolates with *pbp2b* allele D were also ST85, which differs from ST81 at the *ddl* MLST locus. The *pbp2b* and *ddl* genes are near to each other in the bacterial chromosome, and hitchhiking during a recombination event is a recognized phenomenon.^33^ Interestingly, the *pbp2b* allele D sequence and the *ddl* sequence of ST85 are identical to the sequences at the same loci in the PMEN5 clone (Spain^14^-5, ST18), suggesting some shared evolutionary history between the two clones.

Recombination events in the capsular loci can also affect *pbp2x* and/or *pbp1a*, which are located upstream and downstream, respectively, of the capsular biosynthesis operon.^34^ However, there was no evidence for this in the present study, since all 15 isolates with serotypes other than 23F had *pbp2x* and *pbp1a* sequences that were identical to the PMEN1 reference strain.

### Tn916-like ICE-carrying macrolide resistance determinants

During the last two decades, increasing rates of erythromycin resistance among pneumococcal clinical isolates have been reported in many countries. Many *erm*(B)-carrying elements detected in streptococci result from the insertion of *erm*(B) into conjugative transposons of the Tn*916* family, which typically also carry *tet*(M) and confer tetracycline resistance.^10^ Other transposons carrying *mef*(A/E) determinants are also described among pneumococci, but their presence was not analysed in the present study because all erythromycin-resistant PMEN1 isolates included in the study harboured the *erm*(B) gene, and none demonstrated the M phenotype. *erm*(TR) is a rare macrolide resistance determinant among pneumococci; we could not detect any isolates in our collection that possessed this gene.^35^

Nineteen of the 58 isolates were resistant to tetracycline, but susceptible to macrolides; the detection of *tet*(M), *int* and *xis* plus the linkage analysis results suggested the presence of Tn*916* in these isolates (Table 2). Six isolates were PCR-positive for *int* and *xis*, but were susceptible to tetracycline. Loss of tetracycline resistance among PMEN1 isolates was described in France in the 1980s.^36^

**Table 2.** Distribution of Tn*916*-family transposons among PMEN1-like isolates

Resistance pattern (*n*)	Genes detected by PCR (*n*)^a^	Presumed Tn*916*-type ICE	PCR fragment sizes (kb) obtained with specific primers^b^			ST (*n*)	Serotype (*n*)
			J11/J12	tetM2/xis_rv	int_fw/xis_rv		
PEN, TET, CHL, SXT (19)	*tet*(M), *int*, *xis* (19)	Tn*916*	0.9	ND	1.5	ST81 (18), ST85 (1)	23F (16), 19A (2), NT (1)
PEN, CHL, SXT (4)	*int*, *xis* (4)	Tn*916*-like [no *tet*(M) detected]	0.9	ND	1.5	ST85 (4)	23F (4)
PEN, ERY, CLI, CHL, SXT (2)	*erm*(B), *int*, *xis* (2)	Tn*916*-like [no *tet*(M) detected]	0.9	ND	1.5	ST81 (2)	23F (2)
PEN, ERY, CLI, TET, CHL, SXT (32)	*erm*(B), *tet*(M), *int*, *xis* (24)	Tn*6002* (10)	3.6	ND	1.5	ST81 (10)	23F (7), 19F (3)
		unknown (14)	—	—	1.5	ST81 (13), ST85 (1)	23F (11), 19F (3)
	*erm*(B), *tet*(M), *int*, *xis*, *tnpA*, *tnpR* (8)	Tn*3872* (4)	0.9	9.1	1.5	ST81 (4)	19A (3), 23F (1)
		Tn*916* + Tn*917* (4)	0.9	3.8	1.5	ST81 (4)	19A (4)
PEN, ERY, CLI, TET, CHL, SXT, LVX (1)	*erm*(B), *tet*(M), *int*, *xis* (1)	Tn*6002* (1)	3.6	ND	1.5	ST81 (1)	23F (1)

PEN, penicillin; TET, tetracycline; CHL, chloramphenicol; SXT, co-trimoxazole; ERY, erythromycin; CLI, clindamycin; LVX, levofloxacin; ND, not done; NT, non-typeable.

^a^None of the erythromycin-resistant PMEN1 isolates harboured *erm*(TR) or *mef*(A/E).

^b^PCR product size expected for linkage experiments: J11/J12 (0.9 kb for Tn*916* and Tn*3872*, 3.6 kb for Tn*6002* and 7.9 kb for Tn*1545* and Tn*6003*); tetM2/xis_rv (3.8 kb for Tn*917* and 9.1 kb for Tn*3872*); and int_fw/xis_rv (1.5 kb for all Tn*916*-family transposons).

The remaining 33 of 58 isolates were resistant to both erythromycin and tetracycline, and among these 33 isolates multiple combinations of Tn*916*-related genes were found. Sequence alignments and comparisons with known pneumococcal ICEs predicted the presence of different transposons: *Tn*6002 (*n* = 11), *Tn*3872 (*n* = 4) and the combination of *Tn*916 plus *Tn*917 (*n* = 4). *erm*(B), *tet*(M), *int* and *xis* were detected by PCR in the remaining 14 of the 33 isolates; however, the linkage analysis did not identify any known transposon. The region between *int* and *xis* was apparently conserved in length, but no PCR amplicon was obtained between *tet*(M) and *xis* or between regions flanking the *erm*(B) gene (primers J11/J12). This could suggest a DNA reorganization, a divergent sequence in the primer binding regions or a new genetic element; additional sequencing would be required to determine the genetic element(s) present in these isolates.

### pspA alleles

PspA is an important virulence factor that interferes with the fixation of complement C3, and a loss of virulence has been described among PspA mutants.^37,38^ Fifty-three of 58 *pspA* loci were amplified by PCR; despite three attempts, no PCR product was obtained for five isolates. All 53 sequences belonged to *pspA* family 2 (as defined by previously published classifications)^20^ and were captured within four different clades: clade 3, which includes the PMEN1 reference strain (*n* = 50), and clades 1, 4 and 5 (one each). Among sequences of clade 3, seven different alleles were identified, *pspA*^A^ to *pspA*^G^ (see Figure S1, available as Supplementary data at *JAC* Online). The PMEN1 reference strain had *pspA*^A^. In addition, one isolate had a truncated *pspA* gene due to a transposase insertion at bp 957. Two main alleles were detected: *pspA*^A^ (*n* = 17 isolates) and *pspA*^D^ (*n* = 22 isolates; a 243 bp deletion with respect to the *pspA*^A^ allele). The sequence variability of *pspA* in the present study is in agreement with that described previously,^11,20^ supporting the hypothesis that pneumococci may be able to acquire changes in *pspA* to evade host immune defence systems. No significant association between the *psp*A allele and the type of invasive disease or the 30 day mortality was observed among our isolates (data not shown), although the lack of an association could be due to the small sample size.

### pspC alleles

PspC promotes the adherence and invasion of epithelial cells, and PspC mutants showed a reduced ability for nasopharyngeal colonization.^39,40^ Like PspA, PspC is also a candidate for a protein-based pneumococcal vaccine. Forty-six isolates (79.3%) shared the same *pspC* PCR-RFLP pattern (*pspC*^A^) as that of the PMEN1 reference strain, as depicted in Figure 2. Ten additional PCR-RFLP patterns were detected (*pspC*^B^ to *pspC*^K^), nine of which were observed in only one isolate (*pspC*^J^ was observed in two isolates). Both *pspC* and *psp*A failed to amplify for one isolate. Both loci have been identified as recombination hotspots in the PMEN1 genome so the sequence variability noted here is consistent with previous work.^11^

### Analysis of similarities between isolates

**Figure 3.**
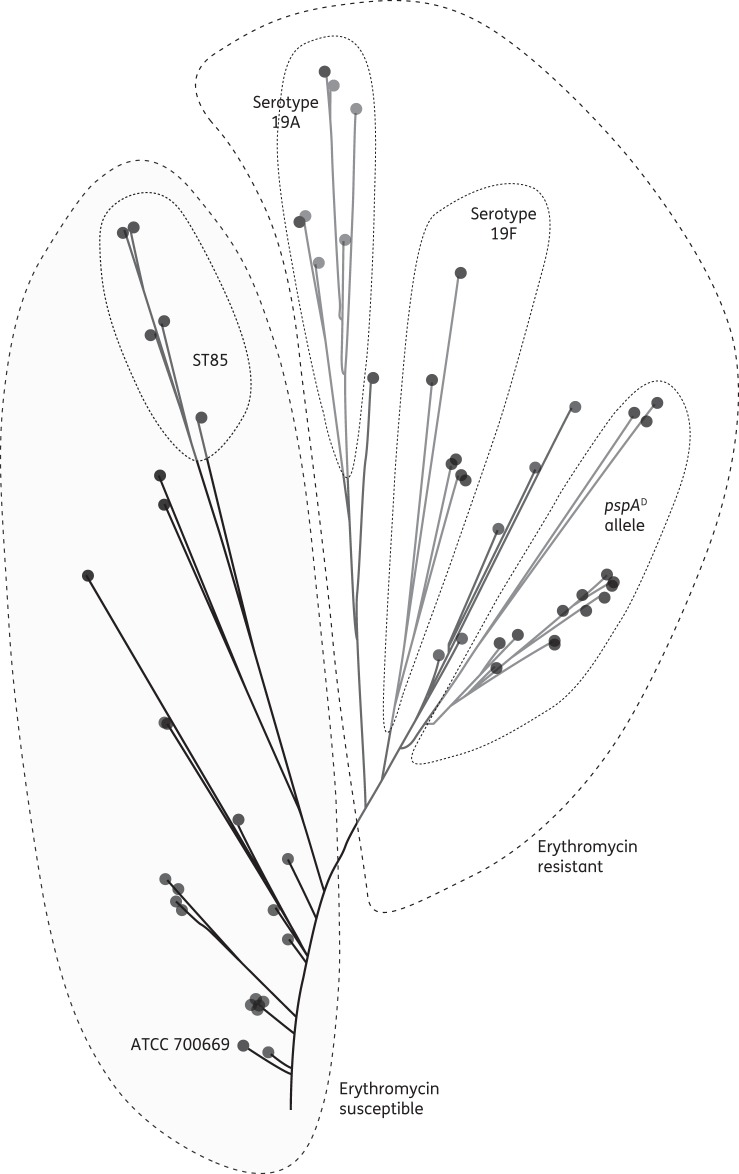
SplitsTree showing the similarity of all 58 PMEN1 isolates. Broken lines separate clusters with erythromycin-susceptible and erythromycin-resistant isolates. Minor clusters are indicated with dotted lines.

Figure 3 shows a SplitsTree analysis of the similarities of the PMEN1 isolates. There were two major clusters of erythromycin-susceptible and erythromycin-resistant isolates, and all PMEN1 isolates included in the erythromycin-susceptible cluster were isolated before 2004. Within these two clusters, four minor clusters were observed: 5 of 6 ST85 isolates, all 6 serotype 19F isolates, 7 of 9 serotype 19A isolates and 16 of 22 isolates with *pspA*^D^; however, there was no association between these clusters and the year of PMEN1 isolation (data not shown).

Although the present study was carried out in a single hospital, this is to our knowledge the first time that the clinical characteristics of adult patients with IPD caused by PMEN1-like isolates have been described. We also characterized loci associated with antibiotic resistance determinants and surface-exposed proteins in the PMEN1 genome of these Spanish isolates and, not surprisingly, noted that these loci have evolved over time. PMEN1 is a well-recognized and globally distributed multidrug-resistant pneumococcal clone and was first recognized in our hospital nearly 30 years ago. Despite having been an important pneumococcal cause of disease in our geographical area during this period, no PMEN1 isolates have been identified among cases of IPD from 2010 onwards, which may be related to the introduction of PCV7 (and a resultant herd immunity) in our adult population. The emergence, dissemination and then possible elimination of such an important clone are interesting biological and evolutionary events.

## Funding

This work was supported by grants from Fondo de Investigaciones Sanitarias de la Seguridad Social (PI 11/00763) and CIBER de Enfermedades Respiratorias, CIBERES (CB06/06/0037), run by the Instituto de Salud Carlos III (ISCIII), Madrid, Spain. A. D. was supported by a grant from Formación de Profesorado Universitario (FPU; Ministerio de Educación, Spain). A. B. B. is a Wellcome Trust Career Development Fellow (ref. no. 083511/Z/07/Z).

## Transparency declarations

C. A., A. F. and J. L. have received funding from Pfizer, unrelated to the present study. All other authors declare no conflicts of interest.

## Supplementary data

Figure S1 is available as Supplementary data at *JAC* Online (http://jac.oxfordjournals.org/).

Supplementary Data
