# Supplementary material for: Evolution and genetic diversity of the Spain23F-ST81 clone causing adult invasive pneumococcal disease in Barcelona (1990–2012)
Source: J Antimicrob Chemother. 2013 Dec 8;69(4):924–31. doi: 10.1093/jac/dkt473 (PMC3956375; doi:10.1093/jac/dkt473)
Supplement: Supplementary Data [file supp_69_4_924__index.html]

Evolution and genetic diversity of the Spain23F-ST81 clone causing adult invasive pneumococcal disease in Barcelona (1990–2012) — Evolution and genetic diversity of the Spain23F-ST81 clone causing adult invasive pneumococcal disease in Barcelona (1990–2012) — Supplementary Data 

# Evolution and genetic diversity of the Spain23F-ST81 clone causing adult invasive pneumococcal disease in Barcelona (1990–2012)

## Supplementary Data

Supplementary Data

**Files in this Data Supplement:**

- Supplementary Data - Doc file
