# Supplementary material for: Evolution and genetic diversity of the Spain23F-ST81 clone causing adult invasive pneumococcal disease in Barcelona (1990–2012)
Source: J Antimicrob Chemother. 2013 Dec 8;69(4):924–31. doi: 10.1093/jac/dkt473 (PMC3956375; doi:10.1093/jac/dkt473)
Supplement: Supplementary Data [file supp_dkt473_dkt473supp.doc]

**Figure S1.** Amino acid substitutions in PspA alleles (from PspA^A^ to PspA^G^) of PMEN1 isolates belonging to clade 3. The PMEN1 reference strain had the PspA^A^ sequence. Shaded regions indicate indels (insertion/deletion fragment).
